# Antisynthetase Syndrome With Paraneoplastic Antibodies

**DOI:** 10.7759/cureus.40354

**Published:** 2023-06-13

**Authors:** Ashbina Pokharel, Ioannis Karageorgiou, Indira Acharya, Tucker Billups, Judith Bateman

**Affiliations:** 1 Internal Medicine, Beaumont Hospital, Royal Oak, USA; 2 Internal Medicine, MedStar Union Memorial Hospital, Baltimore, USA; 3 Rheumatology, Beaumont Hospital, Royal Oak, USA

**Keywords:** autoimmune disease, ductal carcinoma in-situ, antisynthetase syndrome, paraneoplastic syndrome, interstitial lung disease

## Abstract

Antisynthetase syndrome (ASyS) is an uncommon systemic autoimmune disorder characterized by the presence of autoantibodies targeting aminoacyl-transfer RNA (tRNA) synthetase. The syndrome displays a diverse range of clinical manifestations affecting multiple organs, thereby posing a diagnostic challenge. In this report, we present an unusual case of a patient diagnosed with ASyS, displaying positive anti-PL-12 antibodies along with paraneoplastic antibodies. To the best of our knowledge, this is the first documented case in the existing literature describing ASyS with the presence of anti-PL-12 antibodies and concomitant paraneoplastic antibodies in the context of ductal carcinoma in situ.

## Introduction

Antisynthetase syndrome (ASyS) is an autoimmune disorder characterized by a spectrum of clinical manifestations including arthritis, myositis, interstitial lung disease (ILD), Raynaud's phenomenon, fever, and mechanic's hands. The clinical triad of arthritis, myositis, and ILD is observed in approximately 90% of cases [1]^.^ ASyS is associated with the presence of antibodies against aminoacyl-transfer RNA (tRNA) synthetase (anti-ARS), such as anti-Jo-1, anti-PL-7, anti-PL-12, anti-OJ, anti-EJ, anti-KS, anti-Zo, and anti-HA/YRS. A comprehensive approach involving multidisciplinary collaboration between clinical and diagnostic teams is essential for both diagnosis and management. Diagnostic criteria proposed by Connor et al. and Solomon et al. can be utilized to establish the diagnosis of ASyS [2,3]. Treatment approaches are not standardized; however, initial therapy often involves the administration of steroids. Other treatment modalities include cyclophosphamide, azathioprine, cyclosporine, methotrexate, and intravenous immunoglobulin (IVIG). Rituximab is employed in cases of refractory ILD1. The relationship between ASyS and the risk of malignancy has yielded conflicting findings in the literature [4-8]. The objective of this article is to present a challenging diagnostic case of ASyS, elucidate the distinctive clinical features that contributed to the diagnosis, and emphasize the significance of age-appropriate cancer screening for individuals with this syndrome.

## Case presentation

**Figure 1:**
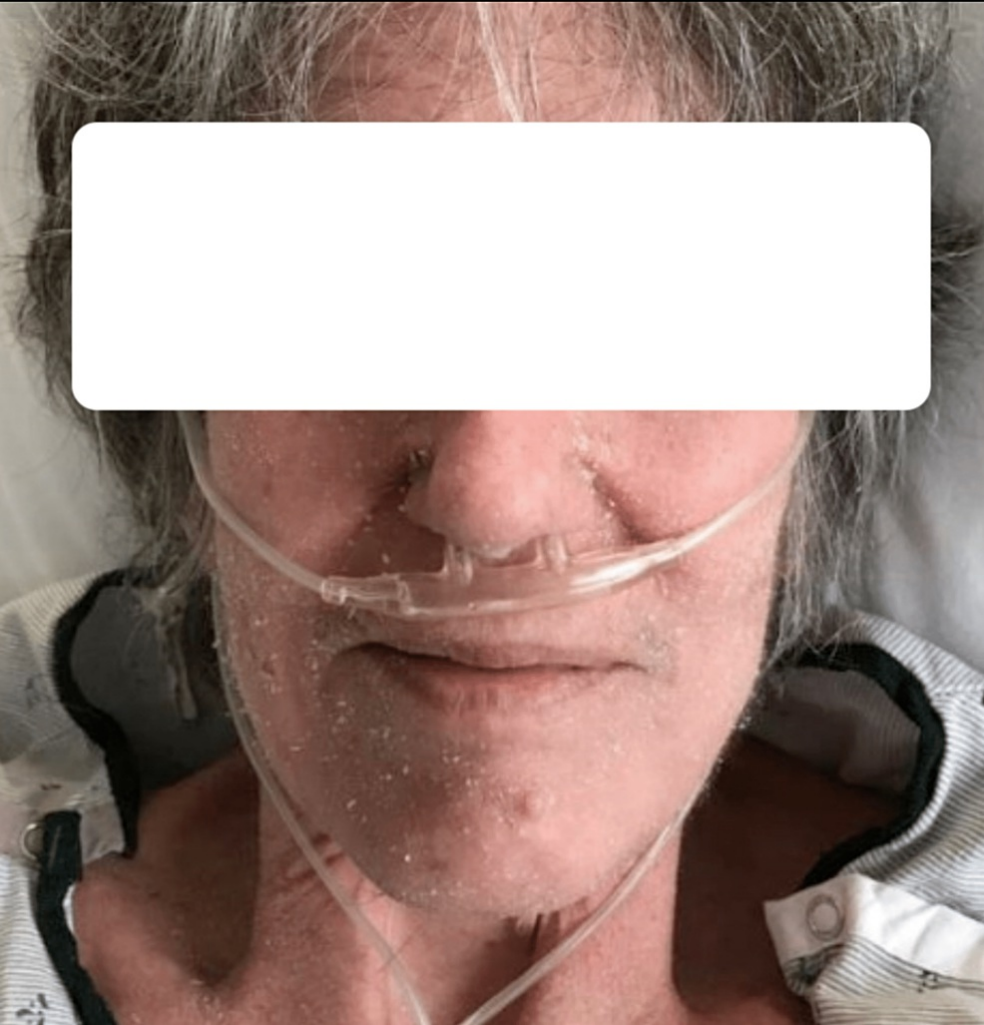
Facial erythematous rash with scaling.

A 67-year-old woman with a medical history of interstitial lung disease (ILD) requiring home oxygen, monoclonal gammopathy of undetermined significance, fibromyalgia, and Raynaud's disease was referred to the emergency department due to worsening shortness of breath over the past three days and a generalized itchy rash persisting for two weeks, as depicted in Figure 1.

The patient had been previously diagnosed with idiopathic pulmonary fibrosis (IPF) two years prior to this current presentation. A chest computed tomography (CT) scan showed usual interstitial pneumonia (UIP) pattern consistent with IPF. Further investigations, including bronchoalveolar lavage cultures, antinuclear antibody, anti-cyclic citrullinated peptide, rheumatoid factor, Sjogren's antibody, and scleroderma antibody, were negative, while human leukocyte antigen B27 (HLA-B27) was positive. The patient experienced adverse effects such as nausea and diarrhea when attempting outpatient treatment with nintedanib or pirfenidone. Pulmonary function tests conducted one year after the IPF diagnosis revealed severe restrictive ventilatory defects. Several months after the IPF diagnosis, the patient developed Raynaud's phenomenon in her fingers, which led to the formation of ulcers.

**Figure 2:**
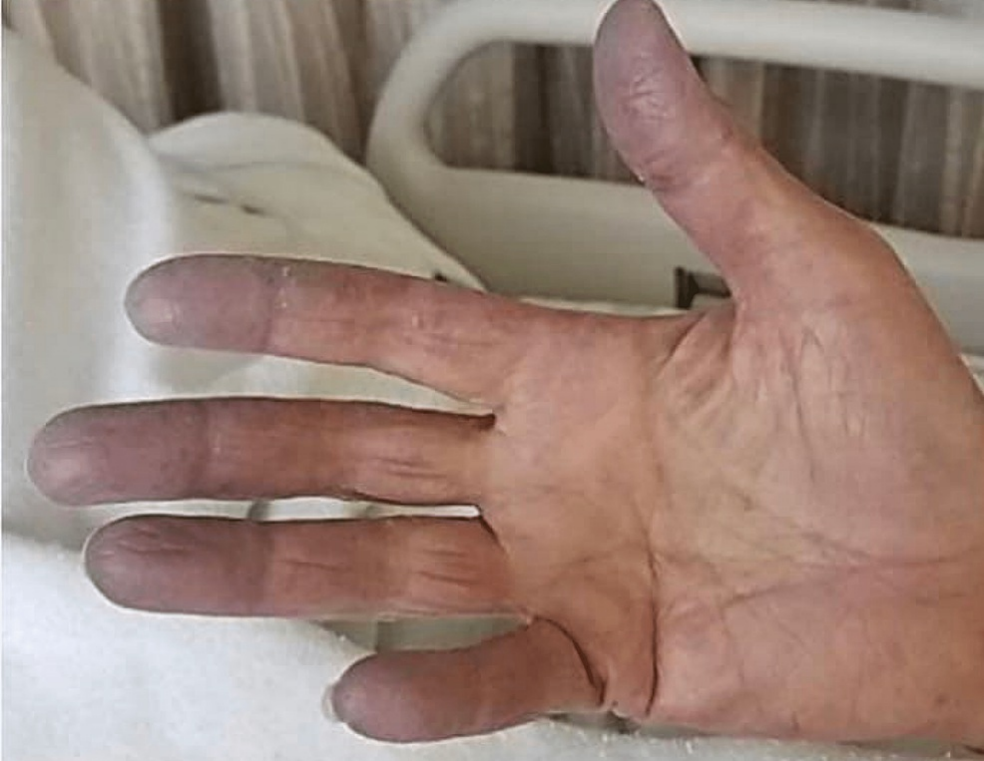
The patient’s right hand with bluish discoloration of fingers, hyperkeratosis, and scaling of the radial aspect of the second finger.

Upon examination, the patient's blood pressure was measured as 131/45 mmHg, heart rate was 100 beats per minute, respiratory rate was 20 breaths per minute, temperature was 36.8 degrees Celsius, and oxygen saturation was 100% on 2 liters of supplemental oxygen. The patient did not exhibit signs of acute distress. During the cardiovascular examination, a soft holosystolic murmur radiating to the axilla was detected, and diffuse inspiratory crackles were heard upon auscultation. A widespread maculopapular erythematous rash with scaling and excoriations was observed on the face, chest, back, posterior neck, arms, and legs. Noteworthy findings also included bluish discoloration of the fingers, hyperkeratosis, and scaling of the radial aspect of the second finger (Figure 2).

Additionally, the patient exhibited reduced proximal muscle strength (3/5) in both upper and lower extremities, along with 2+ bilateral lower extremity edema. The shortness of breath was determined to be secondary to heart failure, and diuretic therapy was initiated, resulting in initial improvement. Initially, the skin rash and itching were suspected to be related to photosensitivity or a drug reaction. Consequently, the patient was treated with diphenhydramine and triamcinolone cream. Initial laboratory investigations revealed leukocytosis with a white blood cell count of 16.7 billion/L (reference range: 3.3-10.7 billion/L), a hemoglobin level of 13 g/dL (reference range: 12-15 g/dL), and a platelet count of 355 billion/L (reference range: 150-400 billion/L). The chemistry panel yielded unremarkable results. Relevant laboratory findings are presented in Table 1.

**Table 1:** Laboratory results on admission IFA: Immunofluorescence assay

Variable	Result	Reference range
Erythrocyte Sedimentation Rate	44mm/hr	0-18 mm/hr
Anti Neutrophil Cytoplasmic Antibody	<1:20	<1:20
Complement C3	76 mg/dL	82-193 mg/dL
Complement C4	23 mg/dL	10-43 mg/dL
Serum Tryptase	5.4 ng/mL	<11.5 ng/mL
Antinuclear antibody	Negative	<1: 160
Anti-double-stranded DNA	21 IU/mL	0-99.9 IU/mL
Anti-Hu antibody, IFA	Detected	Negative
Anti-Yo antibody, IFA	Detected	Negative
Anti-Ri antibody, IFA	Detected	Negative
Creatinine Kinase	749 U/L	30- 150 U/L
Cancer antigen 125	8 U/mL	0-35 U/mL
Carcinoembryonic antigen	2.5 ng/mL	0-3 ng/mL

**Figure 3:**
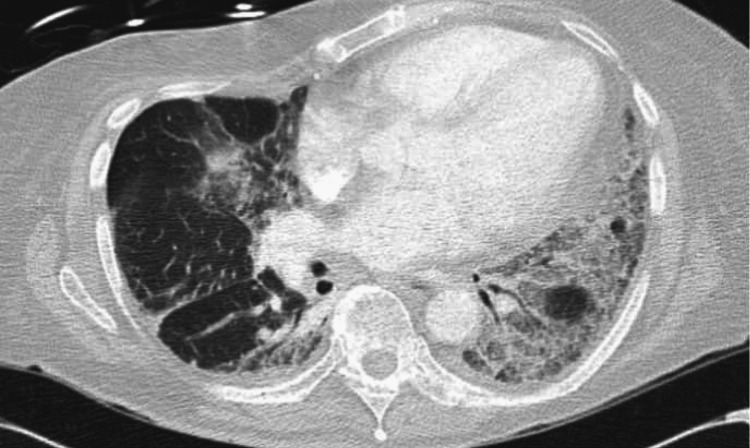
CT chest demonstrating interstitial lung disease which appears slightly worsened with possible developing honeycombing at the bases, superimposed ground glass opacities within the lung bases, right middle lobe, and lingula.

The myositis panel showed a positive result for anti-PL-12 antibodies but was negative for anti-Jo antibodies. Additionally, a CT angiogram of the chest exhibited significant ground glass opacities and honeycombing consistent with ILD (Figure 3). A CT scan of the abdomen and pelvis revealed mild adenopathy in those regions.

Despite treatment, neither the shortness of breath nor the skin lesions showed improvement. Consequently, a skin biopsy was performed, indicating spongiotic dermatitis with eosinophils and neutrophils, but no presence of immune complexes. Electromyography demonstrated signs of inflammatory myopathy. A muscle biopsy of the left thigh displayed lobulated muscle fibers and type 2 fiber atrophy without evidence of inflammation or vasculitis. Consequently, the patient received a diagnosis of antisynthetase syndrome and was treated with intravenous methylprednisolone 60 mg daily during her hospital stay for a duration of 16 days. Upon discharge, she was prescribed prednisone 40 mg daily with a tapering dose of 5 mg per week over a period of four weeks.

During her subsequent rheumatology follow-up appointments, she began taking mycophenolate 3 g daily in conjunction with prednisone 2 g every other day. As part of the oncologic evaluation, an outpatient mammogram was conducted, revealing calcifications in the right breast and right axillary region. A biopsy confirmed grade 2 ductal carcinoma in situ (DCIS) with estrogen receptor positivity (ER+) and progesterone receptor positivity (PR+). Due to the patient's high surgical risk, partial mastectomy was postponed, and treatment with anastrozole was initiated. Subsequently, the patient's steroid dosage was gradually reduced due to osteoporosis, and she continued taking a daily dose of 3 g of mycophenolate.

## Discussion

The disease was initially described by Hochberg et al. nearly three decades ago, highlighting the association between anti-Jo-1 antibodies and interstitial lung disease (ILD) [9]. The diagnostic criteria for this condition were proposed separately by Connors et al. in 2010 and Solomon et al. in 2011 [2,3]. It was in the 2017 European League Against Rheumatism/American College of Rheumatology (EULAR/ACR) annual meeting that ASyS was officially recognized as a distinct disease entity apart from dermatomyositis [10]. However, due to its rarity, there is a lack of validated diagnostic criteria. A recent meta-analysis conducted by Zanframundo et al. focused on the diagnostic criteria for ASyS found in the literature [11]. Most of the included studies incorporated the requirement of antisynthetase autoantibody (ARS) positivity in the diagnostic criteria for ASyS, with most studies necessitating either one ASyS feature plus anti-ARS or multiple ASyS features plus anti-ARS. Only one study did not include anti-ARS positivity and instead required the presence of five ASyS clinical features [11].

The universally agreed-upon clinical manifestations of ASyS include ILD and the evidence of myositis. Additional criteria used to establish the diagnosis encompass arthritis, Raynaud's phenomenon (RP), fever, mechanic's hands, and sclerodactyly [11]. Regarding serological criteria, the presence of antisynthetase antibodies is mandatory [11]. In our case, the patient fulfilled the diagnostic criteria based on both serology, due to positive anti-PL- 12 antibodies, and clinical manifestations, with the presence of ILD, Raynaud phenomenon, myositis, and mechanic's hands.

Due to the infrequency of this condition, the epidemiology of ASyS remains largely uncertain [12-14]. However, the consensus in the literature suggests a higher prevalence of ASyS in women compared to men, with a ratio of 2:1 [12,13]. The median age at disease onset was reported as 52 in a study conducted in Portugal, encompassing an age range of 15 to 75 years [14]. While several studies have indicated a greater frequency and severity of interstitial lung disease (ILD) manifestations in individuals of Black race [5,15], race or ethnicity does not appear to influence the incidence of the disease [13]. Recent advancements have shed light on the molecular and cellular pathways underlying ASyS [12,16-19]. The disease appears to initiate in the lungs of genetically predisposed individuals, where exposure to self-antigens and the breakdown of immune tolerance occur [16]. Consequently, this leads to the activation of the innate and adaptive immune systems, triggering T-cell and B-cell activation as well as the production of autoantibodies [16]. Invasion of other target tissues, where abnormal autoantigen and HLA-I expression occur, results in immune-mediated tissue damage [16].

The defining characteristic of antisynthetase syndrome is the presence of antisynthetase antibodies, which specifically target aminoacyl-tRNA synthetases. These enzymes are responsible for attaching single amino acids to specific tRNAs [12,17-19]. To date, eight subtypes of these antibodies have been identified, namely anti-Jo1, anti-PL- 12, anti-PL7, anti-OJ, anti-KS, anti-EJ, anti-Zo, and anti-Tyr/YRS [12,17-19]. In a retrospective study involving 828 ASyS patients, 72% of them exhibited anti-Jo1 antibodies, while 11.5% had anti-PL-7, 10% had anti-PL- 12, 4.5% had anti-EJ, and 2% had anti-OJ [19]. It is worth noting that these antibodies are typically mutually exclusive, although rare instances of co-positivity have been reported [12]. Notably, co-positivity is often observed with other antibodies, particularly anti-Ro52, which has been associated with an increased risk of cancer and more severe muscle and joint involvement [14]. In our case, the patient tested positive for anti-PL- 12 antibodies but also exhibited positivity for anti-Hu, anti-Yo, and anti-Ri antibodies, which are classically associated with paraneoplastic syndromes [20].

As mentioned earlier, the diagnosis of ASyS requires certain clinical criteria. The classic triad of interstitial lung disease (ILD), myositis, and arthritis is observed in up to 90% of ASyS patients, but it is rare (approximately 20% of cases) for all three symptoms to manifest concurrently during presentation [17]. ILD is the most common extramuscular manifestation of ASyS and serves as a significant prognostic factor [14,15,17]. Poor outcomes are often associated with predictors of more severe lung disease, such as male sex, older age, and black ethnicity [15]. Additionally, the co-occurrence of anti-Jo1 and anti-Ro52 antibodies is linked to more severe and progressive ILD [14]. Other common clinical findings in ASyS patients include Raynaud's phenomenon, mechanic's hands, fever, dysphagia, pulmonary hypertension, and sicca symptoms [11,12]. Interestingly, each antibody type is associated with specific clinical manifestations [14]. For instance, anti-Jo1 positive patients frequently exhibit non-specific interstitial pneumonia (NSIP) pattern ILD, followed by symmetrical polyarticular arthritis and myositis [14]. In the case of anti-PL-12 positive patients, ILD is more prevalent, followed by arthritis [14]. Myositis is less common in this group, while Raynaud's phenomenon is more frequently observed [14]. Anti-PL7 antibody-positive patients also tend to present with ILD and myositis, although arthritis is less common compared to anti-Jo1 patients [14]. Our patient displayed severe ILD, Raynaud's phenomenon, mechanic's hands, and myositis, which aligns with the expected presentation for anti-PL-12 antibody positivity.

The management of ASyS poses a challenge due to the scarcity of randomized controlled trials or high-quality studies investigating different treatment approaches, primarily attributed to the rarity of the disease. Nevertheless, there appears to be a consensus on dividing the treatment into two phases: induction and maintenance [14,17,18]. Induction therapy typically involves initiating systemic corticosteroids, such as prednisone or pulse dose methylprednisolone, which leads to improvement in ILD and myositis in approximately 60% of patients [12,14]. In more severe or steroid-refractory cases, additional immunosuppressive agents like methotrexate, cyclophosphamide, azathioprine, or mycophenolate are recommended [14,17,18]. Cyclophosphamide or intravenous immunoglobulin (IVIG) may be considered for induction treatment in patients with severe ILD or esophageal symptoms, respectively [17,18]. Biological agents such as rituximab have shown success in patients with refractory myositis and ILD [17,18]. For maintenance treatment, commonly used approaches include low-dose steroids (6-12 mg prednisone daily), azathioprine, mycophenolate, or rituximab [14,18]. The optimal treatment duration remains unknown, but most patients undergo a tapering regimen over 12 months [12]. In our case, the patient received pulse-dose steroids for induction therapy and was subsequently maintained on a tapering steroid regimen along with MMF.

The relationship between dermatomyositis, polymyositis, and cancer has been well-established over the years. However, the evidence regarding the predisposition of ASyS to cancer remains unclear [4,7,8,12,14,17,18]. The cancers hypothesized to be associated with ASyS include lung, colorectal, breast, ovarian, and liver cancers [17,18]. Proving or disproving this association is challenging due to the rarity of the antisynthetase syndrome and significant delays in diagnosis. Initial studies suggested a protective effect of ASyS against cancer [4], but subsequent research refuted this notion and highlighted an increased risk of specific cancer diagnoses in patients with certain antibody subtypes, such as anti-Jo112. Notably, one study reported an association between anti-Ro-52 antibodies and a higher prevalence of malignancy [17]. Recent studies, however, indicate no elevated risk of cancer in ASyS patients [18]. Nevertheless, multiple case reports exist in the literature linking ASyS with various types of cancer as mentioned earlier [7,8]. It is important to note that the evidence from these studies is of low quality, as they were retrospective in nature with small patient populations [4,7,8,12,14,17,18]. Therefore, the recommendation remains to conduct age-appropriate cancer screening for all newly diagnosed ASyS patients [12,17]. In our case, age-appropriate cancer screening led to the diagnosis of ER+/PR+ breast cancer in our patient.

## Conclusions

The diagnosis of antisynthetase syndrome (ASyS) poses challenges even for experienced clinicians due to its non-specific clinical manifestations and the lack of synchronicity among all the clinical findings. Therefore, establishing a diagnosis requires a high level of suspicion. This case exemplifies both distinctive and typical features of antisynthetase syndrome. The patient tested negative for anti-Jo-1 antibodies but exhibited positivity for the anti-synthetase antibody anti-PL-12, which is crucial for confirming the diagnosis. Additionally, she presented with an elevated creatine kinase (CK) level, indicative of proximal muscle myopathy. Electromyography (EMG) findings supported the presence of proximal muscle myopathy; however, muscle biopsy did not demonstrate evidence of myopathy. Notably, the patient exhibited positive paraneoplastic antibodies and was diagnosed with estrogen receptor (ER)/progesterone receptor (PR) positive breast cancer, emphasizing the importance of excluding malignancy as a potential underlying cause. Treatment was initiated with pulse-dose steroids and subsequently continued with mycophenolate mofetil (MMF) and low-dose steroids, in accordance with the literature.

## References

[1] Alfraji N, Mazahir U, Chaudhri M, Miskoff J (2021). Anti-synthetase syndrome: a rare and challenging diagnosis for bilateral ground-glass opacities-a case report with literature review. BMC Pulm Med.

[2] Connors GR, Christopher-Stine L, Oddis CV, Danoff SK (2010). Interstitial lung disease associated with the idiopathic inflammatory myopathies: what progress has been made in the past 35 years?. Chest.

[3] Solomon J, Swigris JJ, Brown KK (2011). Myositis-related interstitial lung disease and antisynthetase syndrome. J Bras Pneumol.

[4] Marie I, Guillevin L, Menard JF (2012). Hematological malignancy associated with polymyositis and dermatomyositis. Autoimmun Rev.

[5] Pinal-Fernandez I, Casal-Dominguez M, Huapaya JA (2017). A longitudinal cohort study of the anti-synthetase syndrome: increased severity of interstitial lung disease in black patients and patients with anti-PL7 and anti-PL12 autoantibodies. Rheumatology (Oxford).

[6] Shi J, Li S, Yang H, Zhang Y, Peng Q, Lu X, Wang G (2017). Clinical profiles and prognosis of patients with distinct antisynthetase autoantibodies. J Rheumatol.

[7] Fukui S, Kobayashi K, Fujita Y (2020). Anti-ej antibody-positive anti-synthetase syndrome associated with retroperitoneal sarcoma. Intern Med.

[8] Hara R, Kanazu M, Iwai A (2021). EGFR-mutant lung adenocarcinoma associated with antisynthetase syndrome successfully treated with osimertinib. Thorac Cancer.

[9] Hochberg MC, Feldman D, Stevens MB, Arnett FC, Reichlin M (1984). Antibody to Jo-1 in polymyositis/dermatomyositis: association with interstitial pulmonary disease. J Rheumatol.

[10] Zhao N, Jiang W, Wu H (2022). Clinical features, prognostic factors, and survival of patients with antisynthetase syndrome and interstitial lung disease. Front Immunol.

[11] Zanframundo G, Faghihi-Kashani S, Scirè CA (2022). Defining anti-synthetase syndrome: a systematic literature review. Clin Exp Rheumatol.

[12] Mirrakhimov AE (2015). Antisynthetase syndrome: a review of etiopathogenesis, diagnosis and management. Curr Med Chem.

[13] Wells M, Alawi S, Thin KY (2022). A multidisciplinary approach to the diagnosis of antisynthetase syndrome. Front Med (Lausanne).

[14] Martins P, Dourado E, Melo AT (2022). Clinical characterisation of a multicentre nationwide cohort of patients with antisynthetase syndrome. ARP Rheumatol.

[15] Gasparotto M, Gatto M, Saccon F, Ghirardello A, Iaccarino L, Doria A (2019). Pulmonary involvement in antisynthetase syndrome. Curr Opin Rheumatol.

[16] Gallay L, Gayed C, Hervier B (2018). Antisynthetase syndrome pathogenesis: knowledge and uncertainties. Curr Opin Rheumatol.

[17] Huang K, Aggarwal R (2020). Antisynthetase syndrome: a distinct disease spectrum. J Scleroderma Relat Disord.

[18] Opinc AH, Makowska JS (2021). Antisynthetase syndrome - much more than just a myopathy. Semin Arthritis Rheum.

[19] Cavagna L, Trallero-Araguás E, Meloni F (2019). Influence of antisynthetase antibodies specificities on antisynthetase syndrome clinical spectrum time course. J Clin Med.

[20] Dalmau J, Posner JB (1994). Neurologic paraneoplastic antibodies (anti-Yo; anti-Hu; anti-Ri): the case for a nomenclature based on antibody and antigen specificity. Neurology.

